# Prevailing of HPV-16 and 52 genotype in 2022–2023 in Sanandaj, Iran

**DOI:** 10.1186/s12985-024-02373-3

**Published:** 2024-05-07

**Authors:** Mohammad Haddadi, Leila Atefmehr, Saeed Motlaghzadeh, Fatemeh Hejami, Fatemeh Sadat Elyasi, Negar Zafarian, Zahra Taghiabadi, Amir Aboofazeli, Hadi Yarahmady, Parisa Modaresi, Aniseh Dadgar, Mersede Arbabinia, Mina Naderisemiromi, Sonya Najafpour, Asra Sharifi, Anvar Gholami, Arvin Mamandi, Arash Letafati

**Affiliations:** 1 Research Center for Clinical Virology, Tehran University of Medical Science, Tehran, Iran; 2 Department of Medical Virology, Faculty of Medicine, Iran University of Medical Sciences, Tehran, Iran; 3 Department of Epidemiology and Biostatistics, School of Public Health, Tehran University of Medical Sciences, Tehran, Iran; 4 Department of Infectious Immunology, Manchester University, Manchester, UK; 5 Department of Virology, Faculty of Public Health, Tehran University of Medical Sciences, Tehran, Iran

**Keywords:** HPV, Genotyping, Prevalence, Cancer, Cervical cancer

## Abstract

**Introduction:**

Human papillomavirus (HPV) presents a potential threat to the onset of carcinogenesis in the cervix, anogenital regions, and oropharynx. HPV encompasses over 200 types, with at least 12 having the potential to cause cancer, impacting the majority of sexually active individuals. In this current research, we explore the occurrence and spread of HPV genotypes.

**Material and methods:**

During this cross-sectional study conducted in Sanandaj, Iran from Feb 2022 to Aug 2023, diverse samples including oral, vaginal, and genital were collected from individuals referred to private laboratories in Sanandaj, Iran. After sample collection and DNA extraction (FAVORGEN, Taiwan), they were subjected to PCR and genotyping (MehrViru, Iran). The subsequent statistical analysis unveiled infection rates across different demographics and age groups. STATA (version 17) were used for statistical analysis. We examined infection rates across demographics using t-tests and Odds Ratio.

**Results:**

Overall, 26% (249) out of 950 cases tested positive for HPV, with 69% of these classified as high-risk. Among the examined population, 98% (933) were female, and 2% (17) were male. Females aged 31–40 exhibited the highest percentage of HPV prevalence (115/460) in the study with the majority of positive cases belonging to HR genotypes. The overall most frequent genotypes identified were 6, 16, 52, 53, 51, 58, and 56. HPV-16 exhibited the highest frequency among HR genotypes, accounting for 42 (17%) occurrences, followed by HPV-52 with a frequency of 32 (13%).

**Conclusion:**

Our findings emphasize the significant prevalence of HPV among females, particularly in the 21–30 age group. The identification of high-risk genotypes, underscores the importance of targeted interventions for specific age cohorts. The age-stratified analysis highlights a consistent predominance of high-risk HPV across age groups, indicating the need for age-specific preventive measures. These results contribute valuable information for designing effective screening and vaccination strategies, to alleviate the impact of diseases associated with HPV.

## Introduction

Human papillomavirus (HPV) is recognized as sexually transmitted infection (STI). It carries the potential to induce carcinogenesis in the cervix, anogenital areas, and oropharynx [1, 2]. HPV contributes to nearly 99.7% of cervical cancer cases, ranking among the most widespread cancers in women [3]. In 2020, cervical cancer had a global impact on more than 604,000 individuals (13·3 cases per 100,000 female) and 341,831 deaths (7·2 fatality per 100,000 female), with Asia experiencing the highest prevalence (58%) [4]. Cervical cancer incidence in Iran was reported to 1.90 per 100,000 individuals [5]. In the Iranian female population, prevalence of HPV was detected in 58% of low-grade squamous intraepithelial lesions, 69% of high-grade cases, and 81% of patients with invasive cervical cancer [6].

In addition, HPV is found in over 88% of anal cancers and over 95% of anal high-grade squamous intraepithelial lesions (HSIL), highlighting its major role in anal cancer. Globally, a total of 50,865 new instances of anal cancer were reported in 2020 [7]. In a cross-sectional study (2020–2021) conducted in Tehran, 23.1% of women tested positive for anal HPV [8].

Furthermore, beyond the usual dangers of smoking and alcohol consumption, HPV has appeared as a significant element in oropharyngeal cancer, constituting a distinct subset of head and neck cancers [9]. In a study conducted in Shiraz, Iran, 14% of 100 oral cancer cases were found to have HPV infection [10]. Research shows rise in HPV infection over the past decade in Iran, likely attributed to insufficient sex education for youth and inadequate access to the HPV vaccine [11].

Currently, Over 200 HPV genotypes have been detected [12]. In the context of cancer, HPV types are categorized into two major group which are low-risk HPV (LR-HPV: type 6, 11, 42, 43, 81 and 83) and high-risk HPV (HR-HPV: type 16, 18, 31, 33, 35, 39, 45, 51, 52, 56, 58, 59) [13]. Understanding HPV genotype distribution would be essential for malignancy screening strategies and the development of effective vaccination targeting HPV-related carcinoma [14]. The HPV vaccination program has the capacity to prevent 90% of cervical cancer [15]. To eliminate cervical cancer in lower-middle-income countries, Brisson et al. recommend achieving 90% coverage for girls-only HPV vaccination, undergoing screening twice in a lifetime, and ensuring sustained safeguard against HR-HPV (16, 18, 31, 33, 45, 52, and 58) [16]. Therefore, in this investigation, our objective was to evaluate the frequency of HPV infection divided by genotypes and identify the frequency of HR-HPV genotypes among Iranian females specifically, in Sanandaj, Iran. Our goal is to provide compelling evidence that supports the inclusion of the HPV vaccine in Iran’s national vaccination program.

## Methods and materials

### Study population

The present cross-sectional study was conducted on various samples, including anal swab, anal wart, biopsy of skin lesions, cervical swab, genital swab, paraffin-embedded block of tissue, skin scraping, throat swab, and vaginal swab. The samples were collected from individuals who visited the laboratories of Sanandaj city in collaboration with the Clinical Virology Research Center (RCCV) at Tehran University of Medical Sciences. The research was conducted from Feb 2022 to Aug 2023. Individuals who were included in the survey were invited to complete informed consent. The participating patients did not undergo any medical interventions.

In the standard cytology assessment, physicians forwarded samples, including Pap-Preps (liquid-based cytology) and secretions related to the cervix, to laboratories following established cervical cancer screening procedures. However, for individuals not adhering to the regular screening protocols, such as those who are virgins, samples were obtained with shallower insertion from cervical and vaginal secretions. This collection was performed by either physicians or trained laboratory staff for HPV identification. In the case of males, samples were acquired through anal and penile biopsies and skin scrape sample. The testing procedures on these samples are conducted on the same day as collection, with the samples kept at a temperature of 4 °C until testing is initiated which was a day after sample collection.

The criteria involved women who, following a Pop smear test, chose to undergo additional tests based on the advice of a specialist physician. Furthermore, for women, the criteria extended to those recommended by their healthcare provider to undergo testing due to the presence of malignances including cervical cancer (CC), breast cancer, and Head & Neck Tumor. In the case of men, the criteria included individuals engaging in high-risk sexual behavior, having a sexual partner infected with the HPV, prompting them to seek HPV testing for reassurance about their health status. All demographic information of the patients extracted from laboratory records and subsequently transferred to the RCCV for further analysis.

### DNA extraction and PCR

The pre-amplification of the samples, DNA extraction, and genotyping of HPV were carried out in the Molecular Genetics Department of the laboratory, following the protocol established by the quality control supervisor and under the supervision of RCCV. The extraction of HPV-DNA was carried out using the extraction kit from Favorgen Biotech Corp (FAVORGEN, Taiwan) in accordance with the manufacturer’s instructions. PCR was conducted on the extracted genome by HPV detection kit, and genotyping was conducted by MehrViru’s kit (MehrViru, Iran), equipped to recognize HR-HPV genotypes, including 16, 18, 31, 33, 35, 39, 45, 51, 52, 56, 58, and 59. Additionally, it designates 7 genotypes (26, 53, 66, 67, 68, 73, and 82) as potentially high risk. Moreover, the kit identifies LR-HPV genotypes including 6, 11, 40, 42, 43, 44, 54, 61, 62, 89, and 90.

### Statistical analysis

In this research, we utilized STATA (version 17) for our data analysis. Our approach involved assessing the positive response ratio to the infection test across the entire population, as well as within distinct sex and age categories. Subsequently, we conducted a comparative analysis using the independent t-test. Additionally, Odds Ratio (OR) was considered to evaluate the comparative impact of different groups, maintaining a significance level of 0.05. The individuals who sought medical attention at the specified centers underwent testing for 30 different HPV genotypes. A positive response to the test for one or more types was indicative of infection. The study population was stratified into six age groups, encompassing patients across various age brackets. Following the statistical description of the recorded data, we rigorously examined the reliability and accuracy of our findings through appropriate statistical methodologies.

## Results

### Demographic data

In the examination of HPV prevalence within various demographic subgroups, the data in Table 1 reveals the frequency and percentage of positive and negative cases. Out of 950 cases, 933 (98%) pertain to females, and 17 (2%) to males. The participants were further divided into six age groups for analysis. Within the female subgroup, consisting of 933 cases, 236 (25%) tested positive for HPV, while 697 (75%) tested negative. Among the male participants, 13 out of 17, or 76%, tested positive for HPV. Examining specific age groups, 238 individuals (25%) in the 21–30 age range were tested, with 83 (35%) testing positive and 155 (65%) testing negative. Details for other age groups can be found in Table 2. The highest percentage of positive tests was observed in those under 20 years old (40%) and the 21–30 years old (35%), respectively. However, in the context of numbers of infected individuals, age range between 31 and 40 had more HPV positive cases.

**Table 1:** Frequencies and percent of observation by gender and age

		HPV Positive	HPV Negative	Total
**Gender**	**Female**	236 (25%)	697 (75%)	933
	**Male**	13 (76%)	4 (24%)	17
	**< 20**	2 (40%)	3 (60%)	5
**Age**	**21–30**	83 (35%)	155 (65%)	238
	**31–40**	115 (25%)	345 (75%)	460
	**41–50**	38 (20%)	156 (80%)	194
	**51–60**	9 (20%)	35 (80%)	44
	**61–70**	2 (22%)	7 (78%)	9
	**Total**	249 (26%)	701 (74%)	950

**Table 2:** This table presents the odds ratio of HPV positivity among different age groups, along with their respective *p*-values. In the 21–30 age group, the odds ratio is 2.103 with a corresponding *p*-value of 0.509. For the 31–40 age group, the odds ratio is 1.329 with a p-value of 0.800. In the 41–50 age group, the odds ratio is 1.039 with a p-value of 0.973. For individuals aged 51–60, the odds ratio is 1.029 with a p-value of 0.981. Lastly, in the 61–70 age group, the odds ratio is 1.143 with a p-value of 0.923

	Odds Ratio	***P***-value
**< 20**	–	–
**21–30**	2.103	0.509
**31–40**	1.329	0.800
**41–50**	1.039	0.973
**51–60**	1.029	0.981
**61–70**	1.143	0.923

### Distribution of HPV genotypes

**Fig. 1:**
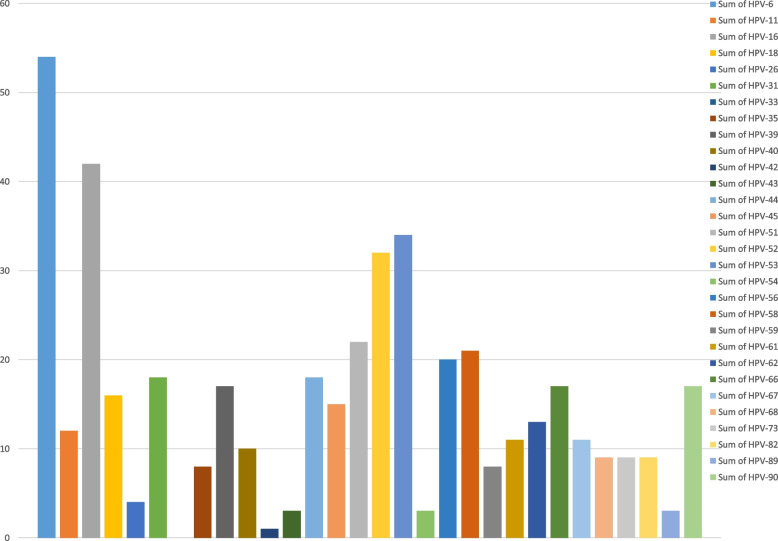
Frequency chart of all HPV genotypes

**Fig. 2:**
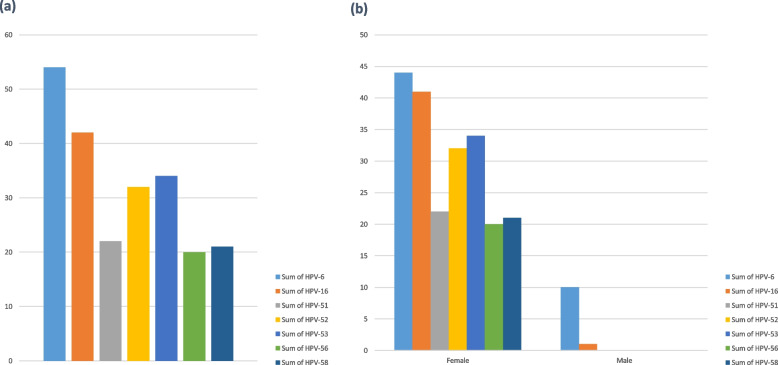
Diagram of the most frequent genotypes including type 6, 16, 51, 52, 53, 56, and 58. **a** In the whole sample, **b** By gender. In part b of this figure, the number of the most frequent genotypes in men and women is shown. Besides, two genotypes of HPV in infected men, which are 11 and 44 genotypes, are not being displayed

**Fig. 3:**
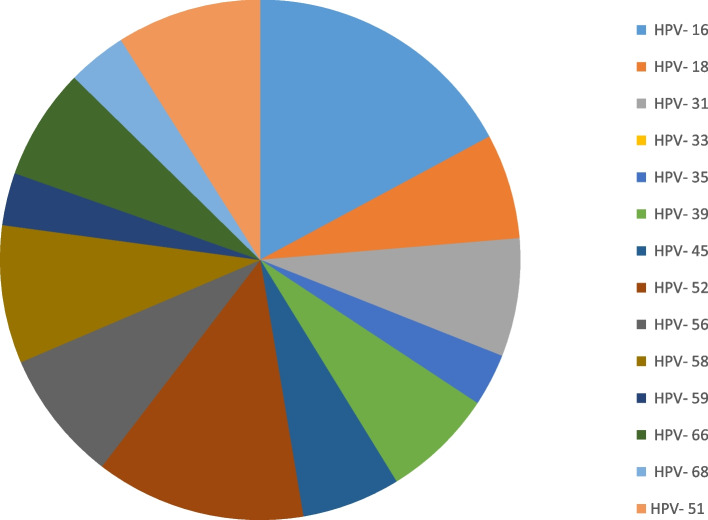
The frequency and distribution of high-risk genotypes. The frequency and percentage for HR genotypes are as follows: HPV-16 with 42 occurrences (17%), HPV-18 with 16 occurrences (6%), HPV-31 with 18 occurrences (7%), HPV-33 with no occurrences (0%), HPV-35 with 8 occurrences (3%), HPV-39 with 17 occurrences (7%), HPV-45 with 15 occurrences (6%), HPV-51 with 22 occurrences (9%), HPV-52 with 32 occurrences (13%), HPV-56 with 20 occurrences (8%), HPV-58 with 21 occurrences (8%), HPV-59 with 8 occurrences (3%), HPV-66 with 17 occurrences (7%), and HPV-68 with 9 occurrences (4%)

The frequency distribution of each genotype can be seen in Fig. 1. The most frequent genotypes are 6, 16, 52, 53, 51, 58, and 56 respectively. The frequency diagram in whole samples and divided by age is shown in Fig. 2. Genotypes 16, 18, 31, 33, 35, 39, 45, 51, 52, 56, 58, 59, 66, and 68 are high-risk types, whose frequency distribution is listed in Fig. 3. Out of total of 236 samples, 164 (69%) HR-HPV and 72 (31%) for LR-HPV detected. The highest frequency in HR genotypes belonged to type 16 with a frequency of 42 (17%), 52 with a frequency of 32 (13%) and 51 with a frequency of 22 (9%), and the lowest frequency is related to types 35 and 59 with a frequency of 8 (3%). In our research, no positive cases of HPV-33 were seen.

### Positive HPV across across sampling types

This section investigates the proportion of positive HPV tests in various sampling categories, including anal, genital, paraffin-based, vaginal, throat and cervical samples. The HPV testing was conducted using different sampling methods, with Table 3 illustrating the frequency distribution. Among the samples studied, cervical samples exhibited the highest number of positive cases (62/393), followed by genital samples (45/68) and anal samples (34/184). Paraffin and skin biopsy samples displayed no negative cases. Vaginal samples had the highest total count (209), comprising 30 positive and 179 negative cases. Statistical analysis revealed highly significant disparities between positive and negative cases across all sample types, with *p*-value ranging from 0.000 to 0.021.

**Table 3:** Frequency distribution table of sampling types for HPV testing

Sample Type	Positive	Negative	Total	Proportion	*P*-Value
**Anal**	34	150	184	0.18	0.021
**Genital**	45	23	68	0.66	0.000
**Oral**	2	0	2	1.00	0.017
**Paraffin**	17	0	17	1.00	0.000
**Vaginal**	30	179	209	0.14	0.000
**Throat**	33	18	51	0.65	0.000
**Cervical**	62	331	393	0.16	0.000
**Biopsy skin**	26	0	26	1.00	0.000
**Sum**	249	701	950	0.26	–

## Discussion

HPV-related disease rates, can differ based on factors like HPV type, geographical situation and sampled body site [17]. Examining the occurrence of HPV infection is essential as it aids in the formulation of strategies for preventing and managing cancer, with a specific focus on cervical cancer [13]. Our study in Sanandaj, located in west of Iran, revealed a 26% prevalence of HPV infection among 950 participants. A 25% prevalence of HPV infection, was identified among 933 females in our research. Our finding aligns with 24% HPV prevalence in 571 healthy women in Iran (2021) [18]. Similarly, a systematic review and meta-analysis conducted between 2004 and 2017 reported a 24.2% pooled prevalence of HPV in Asia, which was lower than the 41.8% prevalence observed in Africa [19]. It’s noteworthy that Iran’s neighboring countries reported varying prevalence rates, with Iraq (17.96%), showing a lower prevalence, while Turkey reported a higher prevalence at 36.3% [20, 21]. Various aspects associated with lifestyle, including sexual behavior, financial status, alertness of disease prevention and screening, as well as the efficacy of laboratory methods and sensitivity of HPV diagnostic techniques, can impact the prevalence rate of HPV in diverse areas [22].

In our study, 13 out of the 17 men suspected of HPV infection tested positive, yielding an infection rate of 76%. However, the worldwide prevalence rate of HPV infection in men was stated to be 31% in a systematic review study [23]. The discrepancy results from the small representation of men (2% of our study population), and the limited sample size may not accurately reflect their prevalence in larger populations. Moreover, higher mortality rates in women attributed to HPV have led to established screening programs, while men’s screening has been less emphasized [24]. Consequently, the notable prevalence of men in our study can be ascribed to their tendency for seek clinical care solely when they manifest clinical symptoms, rather than for routine screening. Nevertheless, in line with our findings, other studies have also reported elevated prevalence rates of HPV in males compared to females [11, 25]. We analyzed HPV prevalence across age groups and found that the greatest incidence of infection occurred in individuals < 20 years (40%), followed by the 21–30 age group at 35%. We observed that the occurrence of HPV infection tends to decrease with age, ranging from under 20 years to 70 years, but it doesn’t completely disappear. In Zhou et al.’s investigation, based on an analysis of age-specified HPV infection, the observed trend revealed a decline in prevalence up to 30 years, followed by an increase until the age of 60 years [26]. Almost mirroring our findings, a study in Malaysia detected the highest HPV prevalence (11.9%) in the 18–24 age range [27], while some other studies reported a different trend with the highest prevalence (7.29%) observed in the 40–49 age group [28]. The elevated prevalence of HPV in female under 20 in our research could be attributed to the lower vaccination percentage between adolescent girls compared to adults. Globally, the HPV vaccination for teenage girls was only 12% in 2021, and it was even lower in South East Asia at 3% [29]. Moreover, among the women aged 19 to 23, there is a higher level of awareness regarding the risk factors of cervical cancer (98.8%), with a corresponding desire for vaccination at 92.5% [30].

Research indicates that testing for high-risk HPV genotypes can serve as an effective initial screening process for cervical cancer, boasting a sensitivity of 93.97% [31]. Therefore, we examined distribution and frequency of different HPV genotype and found that the most commonly encountered genotypes were HPV-6 (54%), then types 16, 52, 53, 51, 58, 56, 31, 44, 90, 39, 66 and 18 in descending order. Likewise, HPV-6, classified as a LR-HPV, demonstrated the highest prevalence in various regions of Iran, registering rates of 50% in the northeast [32], 14.7% in the southeast [33] and 32% in Tehran (among female) [11]. Moreover, our findings revealed that 69% of the participants carried HR-HPV, while 31% had LR-HPV. Shalchimanesh et al. validated our findings with a HR-HPV occurrence rate of 67.12% in Tehran [34], whereas in China out of 414,540 women, HR-HPV was 17.8% [35].

We observed that the predominant LR-HPV genotypes were 6, 44, 90, 62, and 11, while the least prevalent LR genotype was HPV-42. A study in South-Eastern of Iran revealed HPV prevalence of 47% in the HR-group, 34% in the LR-group, and 19% in the potential high-risk group, with HPV-6 being the most predominant LR-HPV (14.7%) [33]. In another study in Eastern of Iran, the most prevalent LR-HPV types were HPV-6, 89, 11, and 42 [36].

In our investigation, the distribution of HR-HPV genotypes revealed varying frequency, with HPV-16 at 17%, followed by HPV-52 at 13%, HPV-51 at 9%, HPV-58 and HPV-56 both at 8%, HPV-31, HPV-39, and HPV-66 each at 7%, HPV-18 and HPV-45 at 6%, HPV-68 at 4%. The lowest frequency of HR-HP was related to HPV-33 (0, 35%), followed HPV-59 and HPV-35 with a frequency of 8 (3%). Global results indicate that HPV-16 (15.56–83.78%) is the most frequently identified HR strain in cancer [37]. In a study across different Iranian provinces, HPV-52 (3.2 and 9.6%) ranked as the second most common HR genotype, consistent with our findings [38]. However, in a distinct region, a different genotype emerging as the second dominant HR types such as HPV-16 in Shanghai [31] and HPV-31 in Hungary [39]. Moreover, HPV-51, a non-vaccine genotype, ranks as the third most frequent high-risk genotype in our study. Hence, recognizing the significant role of HR-HPV types in causing cancer, particularly contributing to 94.1% of squamous cell carcinomas and 83.3% of cervical adenocarcinomas [40], emphasizes the crucial need to prioritize preventive and control measures for this infection. It is essential to acknowledge certain limitations in present study. The absence of random sampling across diverse laboratories may have hindered the ability to draw unequivocal conclusions. The reported HPV prevalence in outpatients may not accurately reflect the actual HPV epidemiology within the entire population. Moreover, we had a limited number of male participants, and given the observed high prevalence of HPV among males in our study, further investigation in a larger target population is warranted. This current research highlighting the clinical development of cervical lesions and the significance of this matter, there is a pressing need for more extensive and comprehensive investigations in the forthcoming.

## Conclusion

Our investigation explored a 26% occurrence of HPV infection and identified its genotypic distribution, revealing that 69% of HR-HPV genotypes were present in Iranian females in Sanandaj, the central city of Kurdistan province in western Iran. The high prevalence of HR subtype of HPV in our findings, underscore the pressing need for proactive measures, advocating for the inclusion of the HPV vaccine in Iran’s national vaccination program. Implementing the HPV vaccination and awareness program, particularly targeting girls under 20 years old who demonstrated a significant prevalence of HPV infection in our study, is a crucial preventive measure. This strategic intervention holds the potential to alleviate the burden of HPV-related diseases such as cancer, fostering improved public health outcomes within the region.

## Data Availability

No datasets were generated or analysed during the current study.
